# Neuroinflammation: an important mechanism of sevoflurane-induced developmental neurotoxicity

**DOI:** 10.3389/fphar.2026.1979399

**Published:** 2026-10-19

**Authors:** Xiaoying Liu, Yue Zhou, Ting Xiang

**Affiliations:** 1 Department of Pharmacy, Xindu District People’s Hospital of Chengdu, Chengdu, China; 2 Department of Nephrology, Institute of Kidney Diseases, West China Hospital of Sichuan University, Chengdu, China

**Keywords:** developmental neurotoxicity, microglial activation, neuroinflammation, NLRP3, sevoflurane

## Abstract

For more than 150 years, the effects of general anaesthetics were assumed to be transient and harmless. However, in the last 2 decades, an increasing number of studies have reported structural and cognitive problems in the brain, particularly the developing brain. Despite increasing evidence of anaesthetic-induced neurotoxicity in animal research, data in humans have been contradictory and confusing. Sevoflurane is the most often used anaesthetic in newborns and applied during millions of procedures worldwide each year; however, this medicine has the potential to be neurotoxic. Short-term or a single exposure to sevoflurane has little effect on cognitive function; however, lengthy or repetitive exposure to general anaesthetics can result in cognitive impairment. This review focuses on the processes through which sevoflurane exposure during development can cause long-term negative consequences for the brain. We focus extensively on the link between sevoflurane anaesthesia and neuroinflammation as critical pathways for sevoflurane-induced developmental neurotoxicity. These findings are intended to offer a theoretical framework for the development of precise neuroprotective techniques and the optimization of current anaesthetic regimens.

## Introduction

Sevoflurane is a commonly used anaesthetic, particularly in paediatric patients. Despite its widespread use, the consequences of sevoflurane exposure on short- and long-term neurologic function remain unknown. Numerous animal studies have reported that morphological and molecular alterations changes in the brain following sevoflurane exposure. Although few human studies have been conducted, their findings remain inconsistent. Indeed, interest in the long-term consequences of sevoflurane exposure in humans has increased in recent years. Two landmark studies, GAS (Neurodevelopmental Outcome at 5 Years of Age After General Anaesthesia or Awake-Regional Anaesthesia in Infancy; 2019) (McCann et al., 2019) and PANDA (Paediatric Anaesthesia Neurodevelopment Assessment; 2016) (Sun et al., 2016), examined the impact of a single sevoflurane exposure on intelligence test scores. Nonetheless, those studies were restricted in scope, leaving plenty of room for further inquiry.

### Pharmacokinetic properties of sevoflurane

The favourable pharmacologic and pharmacokinetic properties of sevoflurane have made it a popular anaesthetic agent, particularly in paediatric patients. At room temperature, sevoflurane is stable, colourless, and not pungent or flammable. It has a distinctive pleasant odour (Apai et al., 2021). Sevoflurane and desflurane have blood gas solubility values of 0.69 and 0.42, respectively, allowing for fast anaesthesia induction and recovery. Sevoflurane has an advantage over desflurane, as it may be used in regular vaporizers (Apai et al., 2021).

A “mammillary” model with five compartments—lungs, vessel-rich organs, muscle, fat close to vessel-rich organs, and peripheral fat—best describes the absorption, distribution, and disposal of volatile anaesthetics (Apai et al., 2021). The solubility of anaesthetics in blood and fatty tissues is inversely related to the rates of induction and recovery. Inhalational anaesthetics have rates of uptake similar to those of continuously administered IV drugs. The alveolar blood concentration (FA) of sevoflurane soon reaches equilibrium with the concentration of inspired sevoflurane (FI), and the FA/FI ratio approaches one. Compared with enflurane and isoflurane, sevoflurane achieves equilibrium faster, and only desflurane and nitrous oxide exhibit more rapid uptake. The FA/FAO ratio measures the clearance of volatile anaesthetics, where the FAO represents the alveolar concentration shortly before anaesthesia is discontinued. Compared with isoflurane and halothane, sevoflurane has a faster rate of elimination, ranking below only desflurane and nitrous oxide.

### Sevoflurane-induced developmental neurotoxicity

Sevoflurane is used for inhalational induction because of its low blood-gas solubility and rapid onset, especially in paediatric anaesthesia. However, animal studies have shown that repeated or extended exposure to sevoflurane is neurotoxic to the developing brain. For example, in mice, maternal anaesthesia with sevoflurane causes social interaction deficiencies in offspring (Chen et al., 2021). Monkeys exposed to sevoflurane anaesthesia in infancy exhibit greater anxiety-related behaviours during adolescence (Raper et al., 2015), and recurrent sevoflurane anaesthesia early in infancy in monkeys causes an anxious phenotype that continues to develop over time (Raper et al., 2018). In mice, neonatal exposure to sevoflurane may increase the risk of cognitive impairment in adulthood (Dai et al., 2020), and repeated prenatal exposure to sevoflurane causes attention-deficit/hyperactivity disorder-like impulsive behaviours in later adulthood (Xie et al., 2020).

Furthermore, a groundbreaking study in 2003 revealed substantial neurodegeneration in many brain areas upon exposure to 0.75%–1.5% isoflurane (Jevtovic-Todorovic et al., 2003). Since then, preclinical investigations of the harmful effects of anaesthetics on the developing brain have dramatically increased (Lin et al., 2017). Animal studies have revealed that exposure to sevoflurane during critical developmental stages might cause neuronal death and demonstrable cognitive impairments. Existing evidence from animal and clinical trials has raised concerns regarding the possible neurotoxic effect of sevoflurane on children, particularly if it interferes with important brain developmental processes. In this review, we investigate the major neuroinflammatory pathways through which sevoflurane affects neurodevelopment.

### Inflammation

Inflammation can directly or indirectly disrupt learning and memory skills by interfering with intracellular signalling pathways in neurons and worsen neuronal damage caused by sevoflurane anaesthesia, promoting the development of developmental neurotoxicity (Useinovic et al., 2022b). Furthermore, sevoflurane anaesthesia may stimulate inflammation-related signalling pathways, leading to increased synthesis of proinflammatory cytokines in the brain, exacerbating neuroinflammatory responses and eventually culminating in age-related cognitive impairment (Cui et al., 2018). This bidirectional vicious cycle is most likely a pathogenic mechanism of sevoflurane-induced neurotoxicity. In recent years, as molecular biology and neuroimmunology studies have advanced, several inflammation-regulating mechanisms mediated by sevoflurane have been gradually elucidated.

The major feature of neuroinflammation is the overexpression of proinflammatory substances resulting from glial activation or immune cell infiltration. Neuroinflammation is the ability of the central and peripheral nervous systems to elicit innate immune responses under pathogenic conditions (Mendiola and Cardona, 2018). Astroglia and microglia are thought to be the main effector cells responsible for mounting inflammatory responses following damage. Microglia are the primary source of cytokines in the central nervous system (CNS) and release proinflammatory cytokines and chemokines such as monocyte chemoattractant protein-1, interleukins, macrophage colony-stimulating factor, tumour necrosis factor (TNF)-α, and macrophage inflammatory protein-1α/β, while astrocytes express receptors for interleukin (IL)-1, IL-8, and IL-6 and macrophage colony-stimulating factor (Kanthasamy et al., 2019). The overexpression of these proinflammatory cytokines and chemokines can lead to neuronal injury (Chen et al., 2018; Takahashi et al., 2008). Repeated sevoflurane exposure in newborn mice activates microglia, releases proinflammatory molecules, and increases the levels of neuroinflammatory factors (TNF-α, IL-8, IL-6, and IL-1β) (Shen et al., 2013a; Xia et al., 2017; Yang et al., 2020). Furthermore, the PI3K/AKT/mTOR pathway in the brains and hippocampi of rats might be involved in sevoflurane-induced developmental neurotoxicity (Wang and Wang, 2019). Moreover, exposing monkeys to sevoflurane when their brains are rapidly growing increases microglial activation, which may be identified by the upregulation of translocator protein expression (Zhang et al., 2016). Maternal exposure to sevoflurane also directly alters foetal glial cells and increases IL-6 levels via phospho-ERK signalling (Hirotsu et al., 2019). Thus, neuroinflammation plays a role in sevoflurane-induced developmental neurotoxicity and may be a relevant target for future investigations (Figure 1).

**FIGURE 1:**
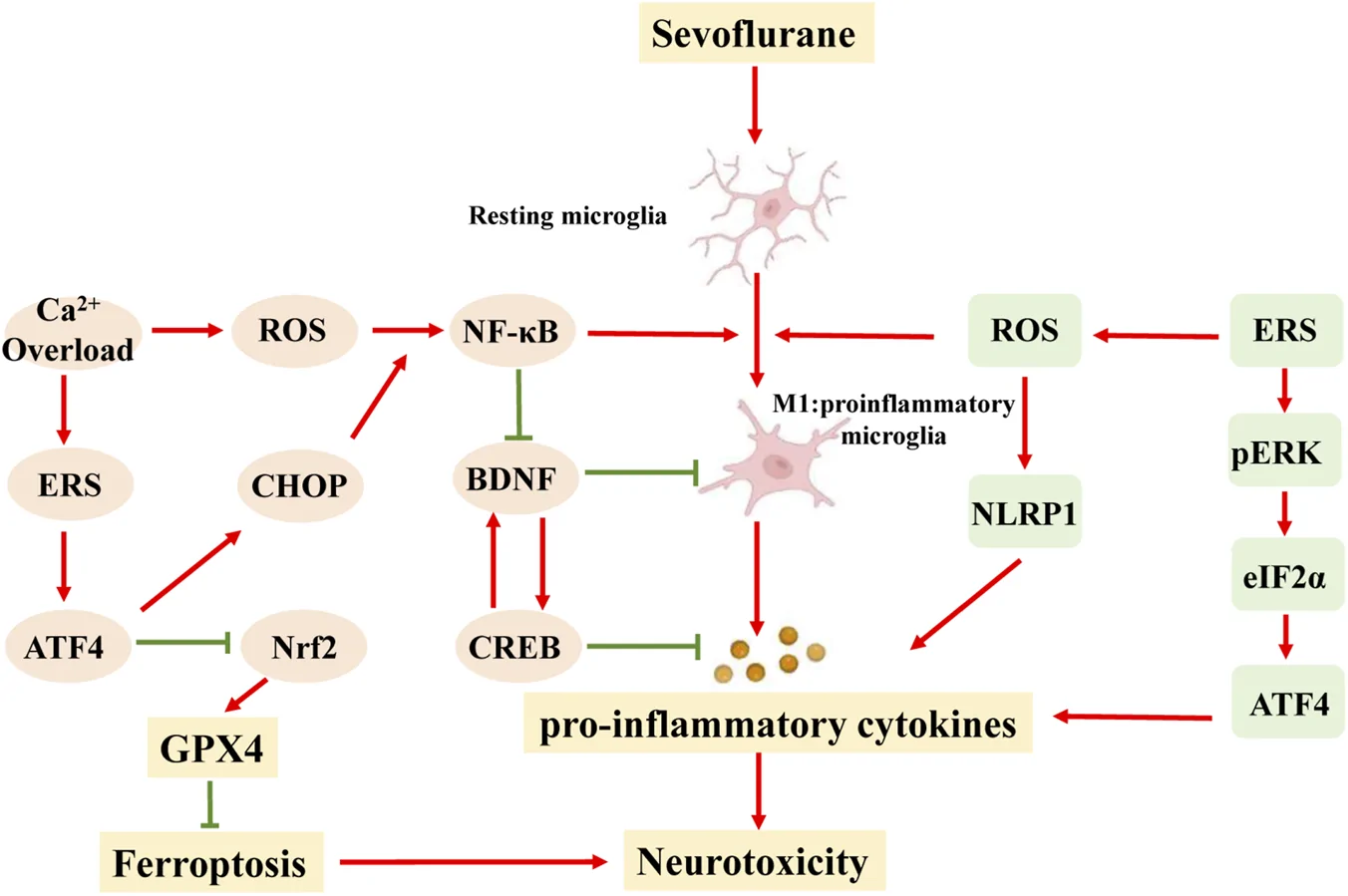
Mechanisms of sevoflurane-induced neurotoxicity. Primary microglia-induced neuroinflammation and ferroptosis were investigated in this study.

## Mechanisms of neuroinflammation

### Microglial activation

Microglia are the primary neuroimmune cells in the human brain and make up 10%–15% of all glial cells (Subhramanyam et al., 2019). Under stable conditions, microglia support neighbouring cells with nutrients and regulate the growth of neurons. Reactive oxygen species (ROS), nitric oxide (NO), and inflammatory substances are released by activated microglia in response to tissue damage or infection (Fodelianaki et al., 2019). Phagocytosis and neurotrophic support facilitate the resolution of inflammation, tissue healing, and remodelling (Li and Barres, 2018). Thus, the microglial phenotype, activation status, and function play major roles in maintaining microglial tissue homeostasis in the central nervous system.

Microglia are specialized resident immune cells in the central nervous system that constantly sample their surroundings via highly dynamic mechanisms to execute immune surveillance activities.

Sevoflurane reduces the inhibitory effects of isatin and PPARγ on hippocampal microglia in aged mice. Moreover, sevoflurane eliminates inhibitory neural signals via endogenous damage-associated molecular patterns (DAMPs) and activates microglia through binding to pattern recognition receptors. Sevoflurane also dramatically increases the release of the endogenous danger signalling molecules HMGB-1 and galectin-3 in hippocampal tissue. These chemicals activate the microglial surface pattern recognition receptors TLR4 and TLR9, which activate the classical MyD88-dependent signalling pathway and PI3K/AKT. These activities increase the phosphorylation level of IκBα, a major regulatory factor of NF-κB, leading to its increased transcription and the release of downstream proinflammatory cytokines, including TNF-α, IL-1β, and IL-6. Delaying the release of HMGB-1 and galectin-3 (Joe et al., 2024) represents a possible target for the prevention and treatment of anaesthesia-related cognitive impairment. In sepsis models, sevoflurane suppresses TLR4 activation, attenuates NF-κB signalling, and has anti-inflammatory effects (Okuno et al., 2019). As a result, sevoflurane may play both protective and harmful roles in neuroinflammation, with effects on inflammatory responses ranging across species and in the context of different underlying illnesses.

### Inflammatory pathways and factors

Studies have demonstrated that sevoflurane can accelerate the migration of microglia, stimulate their activation, and increase their phagocytic activity (Dang et al., 2018). Sevoflurane activates microglia through the NF-κB signalling pathway, leading to increased production of inflammatory markers such as IL-1β, IL-6, and TNF-α through the Notch signalling pathway (Qin et al., 2020), Toll-like signalling pathway (Zhang et al., 2019), AMPK signalling pathway, NF-κB signalling pathway (Han et al., 2019), and other inflammatory signalling pathways. The neuroinflammation resulting from the constant increase in inflammatory cytokine levels may be linked to sevoflurane-induced developmental neurotoxicity.

Moreover, the α7 nicotinic acetylcholine receptor (α7nAChR) is present on both nonneuronal and neuronal cells and is activated upon the binding of acetylcholine (ACh). The α7nAChR activation reduces the nuclear translocation of NF-κB in macrophages, limiting the production of proinflammatory cytokines (such as TNF-α, IL-1β, and IL-6) while concurrently activating the JAK2–STAT3 pathway to promote the expression of anti-inflammatory factors (e.g., IL-10) (Wang et al., 2003). Repeated exposure to therapeutic dosages of sevoflurane decreases acetylcholinesterase expression in the rat hippocampus by increasing ACh catabolism (Yin et al., 2019); in addition, blocking calcium currents decreases presynaptic ACh release (Pavlov and Tracey, 2005), which inhibits α7nAChR activity. The α7nAChR agonist PNU-282987 can counteract this harmful effect (Yin et al., 2019), confirming that sevoflurane-induced cognitive loss is linked to neuroinflammation and suppression of the α7nAChR-mediated anti-inflammatory pathway in rats.

Finally, preclinical evidence suggests that sevoflurane-induced developmental neurotoxicity is caused primarily by neuroinflammatory mechanisms, specifically abnormal activation of the TLR4/NF-κB signalling pathway and the polarization of microglia towards the proinflammatory M1 phenotype. These findings not only reveal the molecular aetiology of sevoflurane neurotoxicity but also serve as a theoretical framework for the development of tailored anti-inflammatory treatment approaches. Notably, researchers have successfully reduced sevoflurane-induced brain injury by targeting these inflammatory signalling pathways, indicating their therapeutic translation potential. In rats, intravenous injection of insulin-like growth factor 1 (IGF-1) restored sevoflurane-suppressed AKT phosphorylation and altered the activity of the PI3K/AKT signalling pathway. In mice, specific deletion of the key hippocampal inflammatory regulator TREM1 decreased microglial M1 polarization and alleviated neuroinflammation and sevoflurane-induced cognitive impairment (Tang et al., 2023). Moreover, morroniside, a traditional Chinese medicine, promotes microglial M2 polarization and inhibits TLR4/NF-κB pathway activation, reducing sevoflurane-induced damage to hippocampal tissue and neuroinflammation. Targeting neuroinflammatory responses is likely to drastically minimize the adverse effects of sevoflurane while preserving the effectiveness of anaesthesia. Thus, efforts to inhibit these inflammatory signalling pathways and the production of proinflammatory cytokines are especially crucial for brain protection in high-risk groups, such as paediatric patients.

### NLRP3 inflammasome activation

Sevoflurane exposure combined with surgical stress induces a multistep neuroinflammatory process characterized by oxidative stress, microglial and astrocytic activation, blood‒brain barrier (BBB) rupture, and eventual neuronal damage (Subramaniyan and Terrando, 2019). The neurotoxic effects are observable at several levels. For example, memory-related behavioural endpoints include delayed escape latency and fewer platform crossings in the Morris water maze test, a decreased discriminating index in the new object identification test, and altered freezing behaviour during fear conditioning. Some studies have also revealed greater anxiety-like behaviours, as reflected by less time spent in the open arm in the elevated plus maze test and less time spent in the centre the open field test. Upstream events that converge on NLRP3 activation in this context include sevoflurane-induced mitochondrial failure, which causes ROS overproduction, intracellular potassium efflux, calcium dysregulation, and poor autophagic clearance of injured organelles (Figure 2). In hippocampal tissue, sevoflurane increases the number of TUNEL-positive neurons; upregulates the expression of NLRP3, cleaved caspase-1, IL-1β, and GSDMD-N; increases the levels of reactive oxygen species and malondialdehyde; and causes mitochondrial swelling and cristae loss. These behavioural and structural changes provide a molecular explanation for the observed sevoflurane-induced cognitive impairments.

**FIGURE 2:**
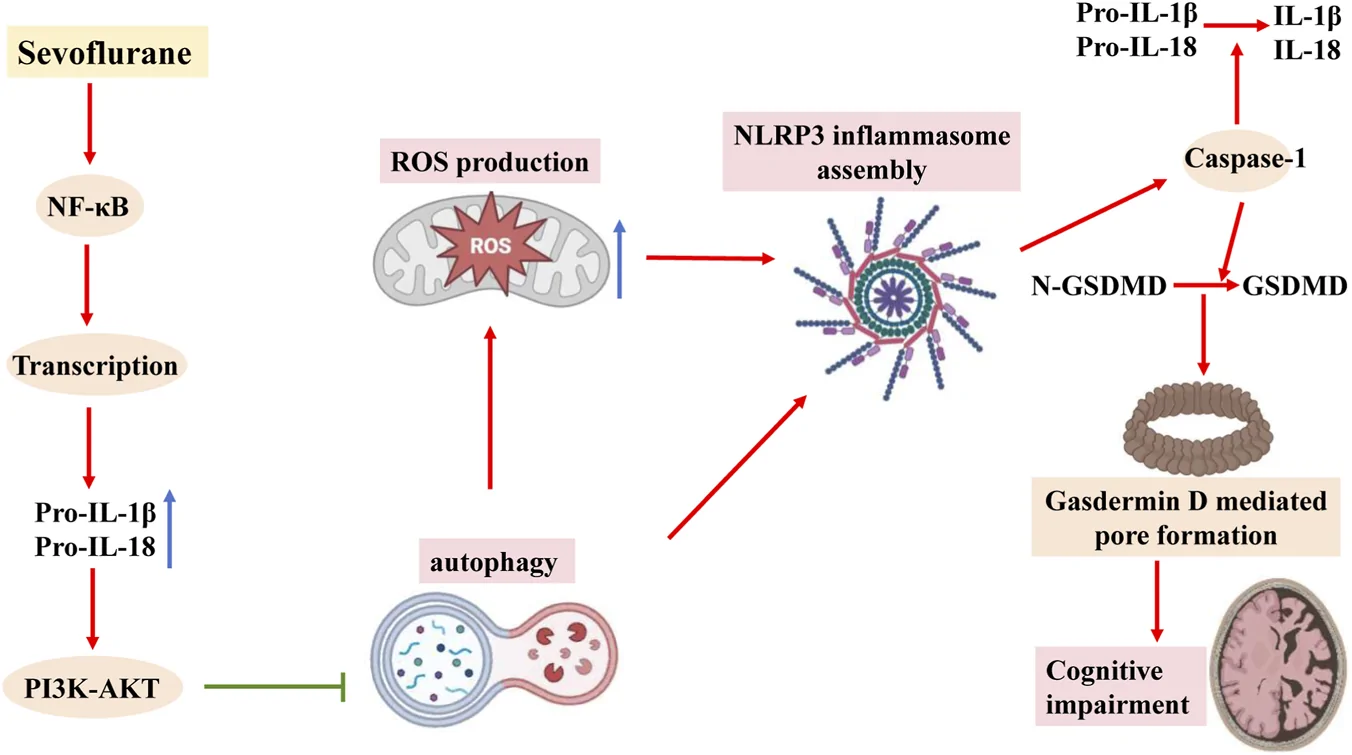
Sevoflurane modulates the NLRP3 inflammasome, which determines neurotoxicity. Sevoflurane activates the NLRP3 inflammasome via upstream triggers such as ROS overproduction, mitochondrial dysfunction, and defective autophagy. This activation results in caspase 1-mediated IL-1β and IL-18 maturation, as well as gasdermin D-dependent pyroptosis, ultimately causing cognitive impairment.

Microglia play a critical role in this process. Sevoflurane exposure has been linked to mitochondrial dysfunction and excessive ROS production, both of which are recognized as upstream signals of NLRP3 inflammasome activation (Su et al., 2025; Subramaniyan and Terrando, 2019). Impaired autophagic flux, as evidenced by a lower LC3-II/I ratio and increased p62 accumulation, is associated with decreased clearance of damaged mitochondria and increased oxidative stress. Furthermore, sevoflurane inhibits histone lactylation, specifically at H3K18. This inhibition downregulates the expression of the m6A reader YTHDF3 and subsequently decreases the translation of the antioxidant enzyme PRDX3, establishing a favourable environment for ROS-driven NLRP3 activation (Zhang et al., 2025). In addition to ROS-mediated priming, other intracellular signalling pathways cooperate to increase NLRP3 activation in microglia. The P2X4R pathway increases ATP-dependent calcium influx and upregulates the expression of the Kv1.3 potassium channel, therefore increasing inflammasome formation. The cGAS‒STING axis is activated when mtDNA leaks into the cytoplasm because of sevoflurane-induced mitochondrial damage, which increases NLRP3 activity (Liu L. et al., 2025; Yang et al., 2024). This mechanism is strengthened by the overexpression of SENP7, which deSUMOylates and activates cGAS (Liu L. et al., 2025). Similarly, extranuclear HMGB1 produced by injured neurons binds to TLR4 and triggers NF-κB signalling, upregulating NLRP3 (Shan et al., 2023). The HDAC6–HSP90/HSP70 axis also affects inflammasome assembly, where sevoflurane-stimulated HDAC6 deacetylates HSP90, altering its interaction with NLRP3 and accelerating the conformational maturation of the inflammasome complex (Lin et al., 2024).

Activation of the NLRP3 inflammasome triggers caspase-1-dependent cleavage of pro-IL-1β and pro-IL-18, leading to a strong inflammatory response. Furthermore, GSDMD induces pyroptotic cell death in microglia and promotes the generation of secondary DAMPs, including HMGB1 and ATP, to sustain neuroinflammation. The release of IL-1β and other proinflammatory cytokines stimulates astrocytes via the p38/MAPK/GSK3β signalling pathway. This activation changes astrocytes into a reactive state characterized by the production of more proinflammatory cytokines and the disruption of their usual supporting roles in neurons, worsening synaptic dysfunction and neuronal damage.

Neurons are not passive targets in this process; they actively engage in inflammasome-mediated disease (Shao et al., 2020; Zheng et al., 2023). Sevoflurane directly activates the NLRP3 inflammasome in neurons, inducing caspase-1-dependent pyroptosis via the NEK7–SNHG3–NLRP3 axis (Ma et al., 2024; Wang et al., 2019). Similar to that in microglia, sevoflurane increases neuronal ROS and malondialdehyde levels, activating the NF-κB pathway and upregulating NLRP3 expression in neurons (Fang et al., 2021; Luo et al., 2023). Sevoflurane also suppresses neuronal autophagy, as evidenced by a decreased LC3-II/I ratio and increased level of p62 (Ye et al., 2019). Furthermore, sevoflurane affects neuronal NLRP3 by inducing histone changes such as deacetylation, which may be reversed by HDAC inhibitors such as SAHA (Liang et al., 2023a). Sevoflurane-induced oxidative stress and NF-κB activation increase neuronal NLRP3 expression, whereas low autophagy owing to SP1/PI3K/AKT/mTOR activation and AMPK/SIRT1 repression leads to mitochondrial damage and chronic ROS signalling (Liu et al., 2021; Lv et al., 2023). Epigenetic pathways also influence these processes. Histone deacetylation inhibits neuroprotective gene expression, whereas YTHDF1-mediated m6A modification stabilizes CREB mRNA and increases CREB/BDNF communication to maintain synaptic resilience (Huang et al., 2023; Ma et al., 2024). Finally, the microRNA-mediated regulation of GPX4 contributes to the epigenetic control of NLRP3 (Qiu et al., 2025). Sevoflurane-induced hypermethylation of the clock gene PER2 inhibits its transcription, reducing AKT pathway activity and activating the NLRP3 inflammasome in microglia (Li and Wang, 2025).

Although there are several routes through which sevoflurane induces developmental neurotoxicity, we believe that neuroinflammation warrants further investigation in the future.

In this study, many innovative approaches and methods in different domains, such as single-cell omics (Wu et al., 2021), proteomics (Qiao and Wang, 2019), metabolomics (Zhang et al., 2020), nanotechnology (Liang et al., 2016), and ultrasound/photoacoustic imaging (Li et al., 2021), have been reported. These new technologies should be used in future research to provide a dynamic, multilevel, multidimensional picture of the molecular pathways underlying sevoflurane-induced developmental neurotoxicity. We believe that the findings of these studies may contribute to improved outcomes in paediatric patients.

### Translational potential: preclinical findings and clinical application

During the critical window of robust synaptogenesis, the prior exposure profile represents a key determinant of neural vulnerability (Zhao et al., 2022). Acute, single exposures induce only transient and modest modifications in dendritic spine density and neuronal function, with no appreciable long-term behavioral consequences in standardized experimental paradigms (Qiu et al., 2024; Qiu et al., 2016). By comparison, anesthetic exposure delivered repetitively or over extended durations can block hippocampal long-term potentiation, potentiate neuroinflammatory activation, and produce age-related deficits in cognitive functions including learning and memory (Shen et al., 2013b; Zhao et al., 2022). Converging evidence from multiple independent datasets highlights a Tau-mediated vulnerability unique to the neonatal brain: sequential phosphorylation of Tau is strongly associated with cognitive impairment following prolonged multi-day anesthetic exposure, whereas single brief exposures elicit only modest neurobehavioral effects (Liang et al., 2023b). Complementary investigations reveal that sevoflurane induces rapid elevations in dendritic spine density within the medial prefrontal cortex during active synaptogenesis. Such structural remodeling may proceed independent of apoptotic signalling yet remains capable of rewiring the assembly of developing neural circuits (Briner et al., 2010). Long-term functional and structural consequences encompass persistent perturbations in dendritic arborization that persist into adolescence following a single anesthetic exposure on postnatal day 7, signifying a lasting imprint on the maturational trajectory of neural structure (Useinovic et al., 2023) (Table 1).

**TABLE 1:** Sevoflurane-induced developmental neurotoxicity in preclinical studies.

Study	Subjects	Anaesthetic dose	Evaluation method(s)	Conclusions
(Makaryus et al., 2018)	Neonatal rats aged 5–35 days	2% Sevoflurane for 6 h	Proton magnetic resonance spectroscopy measurement of the trajectories of N-acetylaspartate; Barnes maze tests for evaluating long-term effects on behaviour	Neonatal rodent brains were more vulnerable to multiple anaesthesia exposures than to a single exposure of the same cumulative duration
(Huang et al., 2017)	Adolescent rats	2% Sevoflurane for 1 h	Expression of Bax and caspase-3	At age 60 days, neuroapoptosis and cognitive dysfunction were detected in rats given repeated doses of sevoflurane (2% for 1 h) at 7 days of age
(Yang et al., 2016)	Early postnatal mice aged ≤42 days	2% Sevoflurane for 6 h	Western blotting and Nissl staining	Sevoflurane exposure during early postnatal life affected the later development of neurons by limiting dendritic cell development
(Xiao et al., 2016)	Rats	3% Sevoflurane for 1 h or 6 h	At age 21 days, synaptic vesicle-associated proteins, dendritic spine density, and synapse ultrastructure; in addition, the effects of sevoflurane treatment on LTP and LTD, two molecular correlates of memory, were studied in CA1 subfields of the hippocampus using electrophysiologic recordings of field potentials in hippocampal slices; neurocognitive performance at 2 months of age using the Morris water maze and novel-object recognition tests	Neonatal rats exposed to 3% sevoflurane for 6 h experienced hippocampus-dependent learning and memory deficits in adulthood; duration of anaesthesia exposure may have different effects on neuronal plasticity and neurocognitive performance; damage increased with increasing duration of anaesthesia
(Chen et al., 2015)	Neonatal rats	1.8% Sevoflurane	Morris water maze test; discrimination learning test	Hippocampal neurogenesis was promoted with a subanaesthetic dose of sevoflurane
(Chung et al., 2015)	Neonatal mice	Sevoflurane for 6 h (3.5% for 90 min, then 3.0% for an additional 90 min, and 2.5% for the last 3 h)	Apoptosis as measured by extraction and Western blotting of brain tissue immediately after sevoflurane exposure; fear tests and behavioural observations were performed at some time after sevoflurane exposure	Sevoflurane-exposed mice exhibited long-term memory impairment and neurodegeneration but not autism-like behaviours
(Liu et al., 2015)	Rhesus monkeys aged 5 or 6 days	2.5% Sevoflurane for 9 h	Frontal cortical tissue assessments immediately after exposure: DNA microarray, lipidomics, Luminex protein analysis, histologic assays	Changes in gene expression, cytokine levels, and lipid metabolism, which led to neuron damage
(Qiu et al., 2015)	Rat neural stem cells	sevoflurane for 0, 3, 6, or 12 h	Levels of the GABAA receptor, Bax, caspase-3, and Bcl-2 after 48 h of culture	With sevoflurane, GABAA activation was inhibited in the CNS, along with apoptosis and degeneration of neural stem cells
(Li et al., 2013)	Rats aged 7 days	1.8% Sevoflurane or 30% oxygen (control) for 4 h	Cerebral cortices evaluated 3 h and 3 days post-sevoflurane exposure; proteomic analysis, Western blotting, apoptosis detection	Sevoflurane may have caused short-term neuronal apoptosis and disturbances in neuronal migration
(Nie et al., 2013)	Rat neural stem cells	Sevoflurane at 0.5×, 1×, or 1.5× MAC for 1 or 6 h	Cell viability, cytotoxicity, apoptosis rate, and proliferation rate 2 days after exposure; a differentiation assay was performed 7 days after exposure	The number of viable neural stem cells and their proliferation increased upon exposure to sevoflurane at 1× or 1.5× MAC for 1 h, but apoptosis was promoted upon exposure to 1× or 1.5× MAC for 6 h; “prolonged sevoflurane exposure may decrease the self-renewal capacity of hippocampal neural stem cells, resulting in cognitive deficits.”
(Satomoto et al., 2009)	Mice aged 6 days	3% Sevoflurane for 6 h	Apoptosis was measured via immunohistochemical staining for activated cleaved caspase-3; Pavlovian conditioned fear test; social recognition and interaction tests	Exposure to inhaled sevoflurane may cause learning deficits and abnormal social behaviours resembling autism spectrum disorder in neonatal mice
(Zhang et al., 2008)	Mice aged 7 days	1.5%, 2.5%, or 3.5% sevoflurane for 1, 2, or 6 h	Electron microscopy; blood glucose level measurements; immunoblotting assays	Subanaesthetic concentrations of sevoflurane can trigger neuronal apoptosis in the postnatal mouse brain

Inhibiting the NLRP3 inflammasome has been shown in preclinical research as a possible mechanism to reduce neurotoxicity caused by sevoflurane. However, owing in large part to pharmacological and safety issues associated with existing inhibitors, this strategy has not yet been implemented clinically. Achieving adequate molecular specificity to reduce adverse outcomes is a major translational hurdle. Despite being successful in preclinical models, numerous potential drugs have shown off-target activity outside of NLRP3. For example, the extensively researched inhibitor MCC950 has shown potential interactions with cardiac potassium channels and possibly hepatotoxicity (Zhou et al., 2023). The need for next-generation analogues with better selectivity for a wider therapeutic window is highlighted by these findings.

The unfavourable pharmacokinetic profile of many inhibitors, especially their limited ability to penetrate the blood–brain barrier, is another significant obstacle. Effective neuroinflammation modulation necessitates sufficient medication exposure in the central nervous system. After systemic treatment, numerous potential drugs, such as certain caspase-1 inhibitors, exhibit low brain bioavailability (Useinovic et al., 2022a); however, their usefulness for treating perioperative neurocognitive problems is thus limited by this limited CNS penetration. Therefore, new delivery methods or chemical changes that improve BBB permeability should be investigated further.

In current developmental pipelines, thorough long-term toxicity studies are still scarce. Immunosuppression and the maintenance of vital host immunity must be balanced in these initiatives. Efficacy is frequently given precedence over thorough toxicity assessment in preclinical research. Given its critical function in innate immune defence, the potential for excessive NLRP3 inflammasome suppression is a major issue (Kelley et al., 2019; Miller et al., 2005). Therefore, generating a dosage schedule that reduces abnormal neuroinflammation without increasing the risk of infection is still an unsolved safety issue (Table 2).

**TABLE 2:** Summary of interventional strategies targeting NLRP3-related neuroinflammation in sevoflurane-induced neurotoxicity.

Therapeutic strategy	Compound and reference	Target(s)	Conclusions
Regulate upstream signalling pathways	Cyclosporine A (Yang et al., 2024)	mPTP/VDAC inhibitors, mitochondrial function	Prevent ROS production and NLRP3 activation in microglia
​	RU5.21 (Yang et al., 2024)	cGAS-STING pathway	Blocks microglial cGAS-STING signalling; inhibits NLRP3 activation
​	Saikosaponin A (Liu et al., 2025a)	HDAC6 signalling	Targets HDAC6 and exerts an immunomodulatory effect
Inhibition of NLRP3 inflammasome assembly	MCC950 (Coll et al., 2019)	NLRP3 NACHT domain (ATPase activity)	Inhibits inflammasome assembly; improves spatial memory in aged POCD mice; hepatotoxicity concerns
Interrupt downstream pyroptosis and inflammatory effects	NSA and NU6300 (Shua et al., 2025)	GSDMD (Cys191)	Blocks caspase-mediated cleavage, inhibits N-terminal oligomerization, or disrupts pore formation

## Conclusion

The benefits of sevoflurane have made it a popular inhalational anaesthetic, particularly in young patients. The low blood‒gas partition coefficient of sevoflurane allows for rapid equilibration between the inspired and alveolar concentrations, leading to faster anaesthesia induction and emergence. These properties allow patients to respond promptly to sevoflurane while minimizing the negative side effects associated with extended systemic exposure.

Nonetheless, sevoflurane is not a perfect agent. More research is needed to determine whether the developing brain is particularly sensitive to anaesthesia. Animal studies have revealed that sevoflurane causes neurotoxicity, as well as eventual learning and memory impairments. Furthermore, frequent or prolonged exposure to sevoflurane may exacerbate these adverse effects in more vulnerable young brains. Thus, additional research is needed to elucidate the underlying processes through which sevoflurane and other anaesthetics cause developmental neurotoxicity.

Finally, sevoflurane activates microglia and increases the production of inflammatory proteins via inflammatory signalling pathways, causing neurons to be injured or killed and increasing the development of associated memory and cognitive disorders. Activated microglia have dual effects on neurons, acting as a toxic or protective agent. Neurons can also be harmed when various functional microglia linkages are destroyed or misaligned. Understanding the effects of sevoflurane on neuroinflammation and the pathophysiology of associated disorders can identify many options for the prevention and treatment of sevoflurane-induced developmental neurotoxicity.
